# Mental Chronic Disease: from psychiatry to neurology

**DOI:** 10.1192/j.eurpsy.2025.1683

**Published:** 2025-08-26

**Authors:** M. Delso González, S. P. Orrego Molina, M. A. Sava, D. Castro Guarderas, M. P. García, A. M. Hualde, C. Terrón, M. D. Manzano Palomo

**Affiliations:** 1 MD Psychiatric Department, Universitary Hospital Infanta Leonor; 2 Head of Nuclear Medicine Department, Universitary Hospital of Getafe; 3 Nuclear Medicine Department, Universitary Hospital Gregorio Marañon; 4 Neurology Department, Nuestra Señora del Rosario Hospital; 5 Neurology Department, Universitary Hospital Infanta Leonor, Madrid, Spain

## Abstract

**Introduction:**

Chronic mental illness is a significant risk factor for developing neurocognitive disorders. Advanced molecular imaging techniques, such as amyloid PET and FDG-PET, provide critical insights into the neurobiological mechanisms that link psychiatry and neurology, enhancing our understanding of the continuum between these fields.

**Objectives:**

This study aims to describe the clinical history of chronic mental illness in a sample of patients with diagnosis of dementia, using molecular imaging to investigate the relationship between psychiatric history and neurodegenerative pathology.

**Methods:**

We conducted a retrospective, descriptive analysis of patients who underwent amyloid PET imaging at the Neurology Department of Infanta Leonor University Hospital from January 2019 to October 2024. Inclusion criteria mandated a documented history of chronic mental illness, irrespective of psychiatric hospitalization. Collected data included demographic variables (age, sex), cardiovascular risk factors, psychiatric diagnoses according to DSM-5, years of mental illness, neurological diagnoses, and results from FDG and amyloid PET imaging. Ethical approval was obtained, and statistical analyses were performed using SPSS 22.0.

**Results:**

A total of 25 patients were included. The main characteristics of the sample are shown in Table 1.

Among those with a chronic mental illness history exceeding ten years (N=8), the diagnostic distribution was as follows: 20% Alzheimer’s disease, 20% Lewy body dementia, 20% major depressive disorder, 10% post-traumatic stress disorder, and 10% indeterminate. Notably, 75% of Alzheimer’s patients and 66.6% of those with Lewy body dementia had a history of major depressive disorder. Patients with frontotemporal dementia often presented with neurocognitive behavioral disorders or obsessive-compulsive disorder. Among four patients with psychiatric hospitalization, only one received a definitive neurological diagnosis (frontotemporal dementia).

**Image:**

**Figure:**
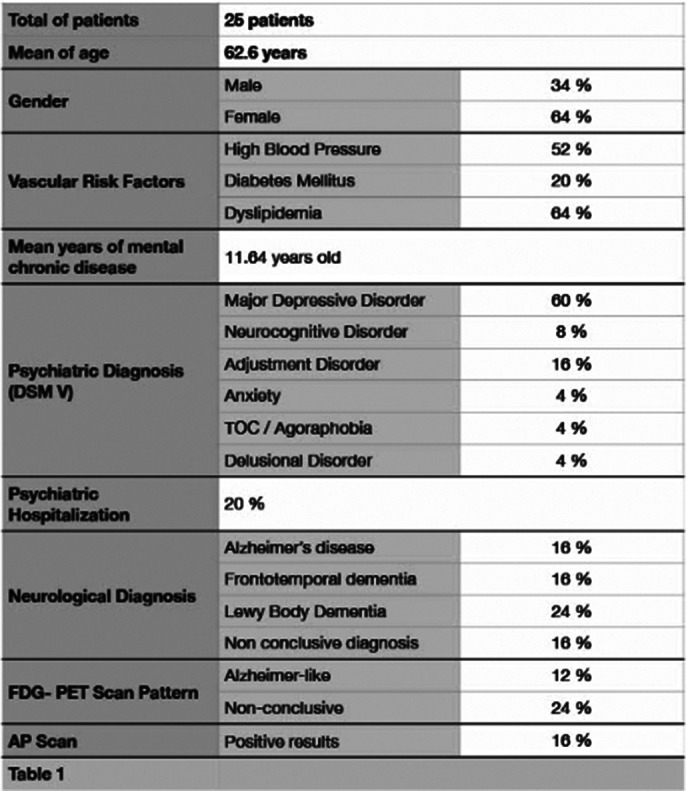

**Conclusions:**

These findings highlight the importance of considering chronic mental illness histories in the cognitive assessment of patients. The neurobiological links between depressive disorders and Alzheimer’s disease emphasize the need for interdisciplinary approaches in clinical practice. Molecular imaging serves as a pivotal tool in refining diagnostic accuracy, complementing both psychiatric and neurological perspectives, and enabling therapeutic strategies to improve patients’ quality of life.

**Disclosure of Interest:**

None Declared

